# M2 macrophage-derived exosomes promote the malignant progression and glycolysis of cervical cancer via Msi1

**DOI:** 10.1186/s41065-026-00703-9

**Published:** 2026-06-25

**Authors:** Liting Sun, Abudureyimu Tunishahan, Xiaowen Li

**Affiliations:** Department of Gynecological Malignancy Radiotherapy, The Affiliated cancer Hospital of Xinjiang Medical University, Urumqi City, Xinjiang 830011 China

**Keywords:** Cervical cancer, M2 macrophages, Exosomes, Musashi1, Glycolysis

## Abstract

**Background:**

Cervical cancer is a common gynecologic malignancy characterized by high recurrence and metastasis rate. Exosomes derived from M2-polarized tumor-associated macrophages play a key role in promoting tumorigenesis and cancer progression. Additionally, musashi1 (Msi1) is a key oncoprotein across multiple malignancies. Therefore, this study aims to determine the role of M2 macrophage-derived exosomal Msi1 in cervical cancer progression and glycolysis and to clarify the underlying mechanism.

**Methods:**

M2 macrophages were identified using microscope, flow cytometry, and western blot. Gene mRNA and protein expression levels were analyzed via quantitative real-time polymerase chain reaction (qRT-PCR) and western blot. The cell proliferation, invasion, apoptosis, and sphere formation were assessed using 5-Ethynyl-2’-deoxyuridine (EdU) staining, Transwell, flow cytometry, and sphere formation assay, respectively. The glucose uptake and lactate production were determined using commercial kits. Exosomes were identified using western blot, transmission electron microscopy, and nanoparticle tracking analysis (NTA). Cellular uptake of exosomes was assessed by incubating SiHa and Ca Ski cells with PKH67-labeled exosomes. Cervical cancer xenograft mouse models were established to analyze the role of M2 macrophage-derived exosomal Msi1.

**Results:**

Msi1 expression was increased in M2 macrophages (**P < 0.01 and ***P < 0.001). Functionally, M2 macrophages enhanced cervical cancer cell proliferation, invasion, aerobic glycolysis, and stemness as well as suppressed cell apoptosis through secreting exosomes (*P < 0.05, **P < 0.01, and ***P < 0.001). Importantly, Msi1 expression was increased in M2 macrophage-derived exosomes (***P < 0.001). Mechanically, M2 macrophage-derived exosomes promoted malignancy and glycolysis by carrying Msi1 (*P < 0.01 and ***P < 0.001). In vivo, M2 macrophage-derived exosomal Msi1 accelerated cervical cancer progression (***P < 0.001).

**Conclusion:**

M2 macrophage-derived exosomes promote cervical cancer progression and glycolysis by delivering Msi1 to recipient cells.

**Graphical Abstract:**

**Figure:**
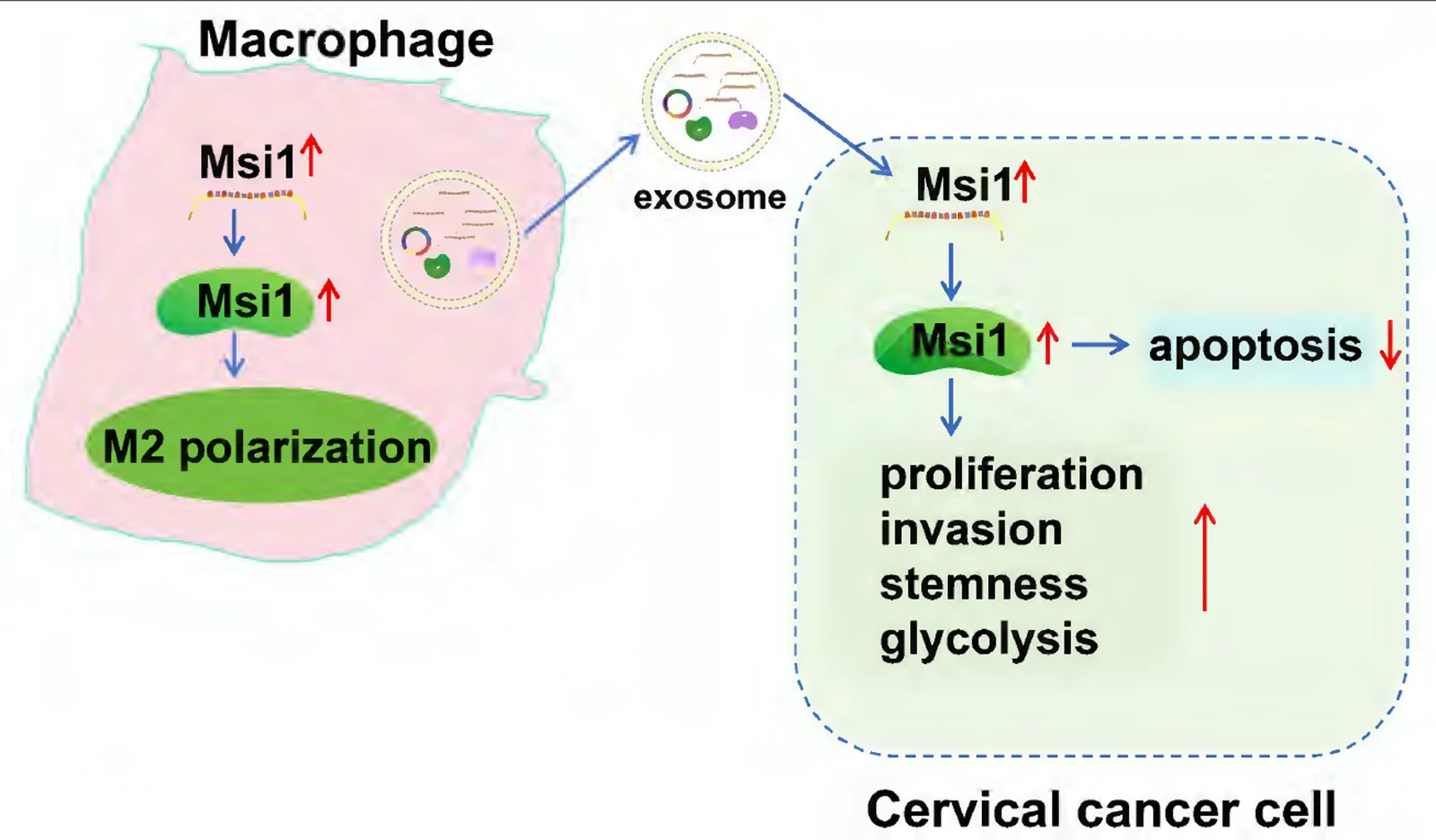

**Supplementary Information:**

The online version contains supplementary material available at https://doi.org/10.1186/s41065-026-00703-9.

## Highlights

Msi1 expression is elevated in M2 macrophages and M2 macrophage-derived exosomes.

M2 macrophages promote cervical cancer progression and glycolysis partially through exosome secretion.

M2 macrophage-derived exosomes promote cervical cancer progression by delivering Msi1.

M2 macrophage-derived exosomes require Msi1 to enhance glycolysis in cervical cancer cells.

M2 macrophage-derived exosomal Msi1 accelerates cervical cancer progression *in vivo*.

**Supplementary Information:**

The online version contains supplementary material available at https://doi.org/10.1186/s41065-026-00703-9.

## Introduction

Cervical cancer is a predominant gynecological malignancy that poses a significant threat to women’s well-being [1]. Its development is primarily driven by persistent human papillomavirus (HPV) infection, with additional contributing factors such as smoking and early sexual activity [2–4]. Recent studies have further elucidated the molecular heterogeneity of cervical cancer and the critical role of the tumor microenvironment in treatment resistance [5, 6]. Various treatment strategies are employed for cervical cancer, including radiotherapy, chemotherapy, immunotherapy and surgery [7, 8]. Although advances in these therapeutic modalities have improved the prognosis of cervical cancer, it remains a life-threatening malignancy worldwide [9, 10]. Therefore, further investigation into the pathogenesis of cervical cancer is crucial.

M2 macrophages suppress antitumor immunity, thereby creating a microenvironment that supports tumor development [11]. Persistent HPV infection and cervical cancer progression are closely linked to immune dysregulation within the microenvironment. With the progression of cervical lesions, the infiltration of M2 macrophages progressively increases [12, 13]. Moreover, cervical cancer-derived mesenchymal stromal cells interact with tumor cells to promote M2 macrophage polarization, which further enhances the immunosuppressive tumor microenvironment [14]. Exosome release represents a key functional mechanism of macrophages [15]. Exosomes are 30–150 nm extracellular vesicles that transport proteins and nucleic acids, and other bioactive molecules, playing a pivotal role in communication between cancer cells and the microenvironment [16, 17]. M2 macrophage-derived exosomes have been shown to facilitate hepatocellular carcinoma metastasis by increasing vascular permeability [18]. Additionally, exosomes derived from tumor cells transfer the TIE2 protein to macrophages, thereby promoting angiogenesis in cervical cancer [19]. However, the potential role of M2 macrophage-derived exosomes in cervical cancer initiation and progression has remained unclear.

Musashi1 (Msi1), an RNA-binding protein, is a key regulator of tumorigenesis and cancer progression across multiple malignancies. For example, Msi1 regulates extracellular matrix degradation via the Tissue Inhibitor of Matrix Metalloproteinase 3/Matrix Metalloproteinase 9 pathway, thereby promoting breast cancer metastasis [20]. Huang et al. found that Msi1 promoted prostate cancer cell proliferation, migration and glycolysis via activatingα/β-hydrolase domain 2 (ABHD2) transcription [21]. Msi1 facilitated cancer stem cell properties of glioblastoma cells via upregulating YTH N6-methyladenosine RNA binding protein 1 [22]. Msi1 suppressed the BAK expression via the AKT signaling cascade, thereby inhibiting apoptosis in cervical cancer cells [23]. Msi1 also targeted the cell cycle regulators p21, p27, and p53, thereby promoting the growth of cervical tumors [24]. Furthermore, in cervical cancer, Msi1 enhanced tumor progression through epithelial-mesenchymal transition [25]. Nevertheless, the role of Msi1 in cervical cancer remains largely unknown. Therefore, this study sought to determine the function of M2 macrophage-derived exosomal Msi1 in cervical cancer progression.

## Materials and methods

### Cell culture

The cervical cancer cell lines Ca Ski and SiHa, along with human myeloid leukemia mononuclear cells (THP-1), were obtained from Procell (Wuhan, China). These cells were cultured in RPMI-1640 medium, also supplied by Procell, supplemented with fetal bovine serum (FBS) from the same company.

THP-1 cells were induced to differentiate into M0 macrophages (THP-1/M0) following a 48 h treatment with 100 ng/mL Phorbol-12-myristate-13-acetate (PMA) (Sigma-Aldrich, St. Louis, MO, USA). To polarize the cells into M2 macrophages (THP-1/M2), they were incubated with 20 ng/mL of IL-4 and IL-13 (Peprotech, Suzhou, China) for 48 h. Finally, the M0 and M2 macrophages were verified through microscopy, flow cytometry, and quantitative real-time polymerase chain reaction (qRT-PCR). For the THP-1/M2 + GW4869 (exosomal inhibitor) group, M2-type macrophages were pretreated with 10 µM of GW4869 (Sigma-Aldrich) for 8 h to inhibit exosome production.

Cells were collected to assess cell functions after SiHa and CaSki cells were co-cultured with THP-1/M0, THP-1/M2, or THP-1/M2 + GW4869.

### Isolation and identification of exosomes

Exosomes were isolated from THP-1/M0 and THP-1/M2 cell culture media using the BeyoExo™ Enhanced Exosome Isolation Kit from Cell Culture Media by Precipitation Method (Beyotime) according to manufacturer’s instructions. Since the precipitation-based separation method may simultaneously separate out non-exosome components, a rigorous identification was carried out in this study. To identify the exosomes, the exosome morphology was observed using transmission electron microscopy. Additionally, the size distribution and concentration of isolated exosomes were determined using nanoparticle tracking analysis (NTA). Additionally, CD81, CD9, tumor susceptibility gene 101 (TSG101), and Calnexin protein expression levels were analyzed by western blot. The absence of Calnexin expression and the presence of CD81, CD9, and TSG101 confirmed exosome enrichment. Cellular uptake of exosomes was assessed by incubating SiHa and Ca Ski cells with PKH67-labeled exosomes (Sigma-Aldrich) for 24 h, followed by fluorescence imaging.

For all functional experiments, exosome input was normalized by equal particle number (1 × 10⁹ particles/mL) based on NTA quantification. THP-1/M0 or THP-1/M2-derived exosomes (M0-Exo or M2-Exo) were used to treat SiHa and CaSki cells. Subsequently, cells were collected to analyze the Msi1 expression. Furthermore, THP-1/M2 macrophages were transfected with short hairpin RNA (sh)-NC or shRNA targeting Msi1 (sh-Msi1), after which exosomes were extracted (M2-sh-NC-Exo or M2-sh-Msi-Exo). Next, SiHa and CaSki cells were treated with M2-sh-NC-Exo or M2-sh-Msi-Exo and then collected to assess cell functions. sh-Msi1 and negative control sh-NC were designed and synthesized by RiboBio (RiboBio Inc., Guangzhou, China). Cell transfection was performed utilizing Lipomaster 3000 Transfection Reagent, which was sourced from Vazyme (Nanjing, China).

### Cell proliferation detection

SiHa and Ca Ski cells subjected to different treatments were collected and then reacted with 5-Ethynyl-2’-deoxyuridine (EdU) working solution (10 µM) for 2 h at 37℃. Subsequently, the cells were fixed with 4% paraformaldehyde (Beyotime, Nanjing, China) at 37℃ for 15 min. The fixed cells were placed in permeating liquid and incubated at 37℃ for 0.25 h. The samples were reacted with Click reaction solution in a dark environment at 37℃ for 0.5 h. Cell nuclei were stained with DAPI (MCE, Kennyworth City, NJ, USA) and then the results were observed and captured using fluorescence microscope. BeyoClick™ EdU Cell Proliferation Kit with AF488 was provided by Beyotime. Each group was performed in triplicate.

### Detection of cell invasion

After the 24-well Transwell top chamber was pre-coated with Matrigel, the SiHa and Ca Ski cells with treatments were plated into the 24-well Transwell top chamber and grown in medium without FBS. The medium with FBS (10%) was added into bottom chamber. Subsequently, the invaded cells were fixed using 4% paraformaldehyde (Beyotime) at 37℃ for 0.25 h. Following staining of the cells with 0.1% crystal violet (Zai Kang Biotechnology Co., Ltd., Shanghai, China), image acquisition was performed using a microscope. Each experiment had three duplicates.

### Flow cytometry

Polarization of M2 macrophages was examined. In short, THP-1 cells, THP-1/M0 macrophages, and THP-1/M2 macrophages were collected and incubated with anti-CD11b (ab8878, 1:5000, Abcam) or anti-CD206 (ab192847, 1:100, Abcam) for 45 min at 4℃. After washing, the positive percentage of CD11b and CD206 were analyzed using flow cytometry system. Each experiment had three duplicates.

After collection, cells were washed with phosphate buffer saline (PBS) (Zai Kang Biotechnology Co., Ltd.) and centrifuged for 5 min. The cell pellet was resuspended in Annexin V-FITC binding buffer, followed by staining with Annexin V-FITC and propidium iodide (PI) for a 20-min period in a dark setting. Subsequently, cell apoptosis was assessed utilizing a flow cytometry system. Each experiment had three duplicates.

### Sphere formation

Different SiHa and Ca Ski cells (2 × 10³) were plated in 6-well ultralow-density culture plates (Corning Inc.). Cells were grown in serum-free RPMI-1640 medium (Procell) supplemented with 20 ng/mL epidermal growth factor, 0.5% bovine serum albumin, B27 (1:50) (Sigma-Aldrich). After 14 days of culture, spheroid formation was assessed, and representative imaging was performed with an inverted microscope. All assays were repeated three times.

### Acquisition of clinical samples

The tissue samples were collected from patients who underwent surgery for cervical cancer and participants signed the informed consent form. The experiments were allowed by Ethics Committee of The Affiliated Oncology Hospital, Xinjiang Medical University. The tissue samples were used to analyze the Msi1 mRNA and protein expression. The ROC curves were used to discriminate two distinct groups of Msi1 mRNA expression. All assays were repeated three times.

### qRT-PCR

FastPure Complex Tissue/Cell Total RNA Isolation Kit (Vazyme) was performed to extract the total RNA. Following that, total RNA was reverse-transcribed into cDNA utilizing the Transcriptor First Strand cDNA Synthesis Kit (Roche, Vilvoord, Brussel, Belgium). Finally, the gene mRNA expression was examined by BeyoFast™ SYBR Green qPCR Mix (2X) (Beyotime). The sequence of qRT-PCR reaction was shown in Table 1. Each experiment had three duplicates.

**Table 1:** Primers sequences used for RT-qPCR

Name		Primers for RT-qPCR (5’−3’)
Msi1	Forward	TTGGCAGACTACGCAGGGG
	Reverse	AAGTGACGAAGCCGAAACCC
Arg1	Forward	ACTTAAAGAACAAGAGTGTGATGTG
	Reverse	CATGGCCAGAGATGCTTCCA
IL-10	Forward	ACACATCAGGGGCTTGCTC
	Reverse	GAAGTGGGTGCAGCTGTTCT
TGF-β1	Forward	GGAAATTGAGGGCTTTCGCC
	Reverse	CCGGTAGTGAACCCGTTGAT
β-actin	Forward	CTTCGCGGGCGACGAT
	Reverse	CCACATAGGAATCCTTCTGACC

### Detection of protein expression levels

After the total protein was extracted, the concentration of protein was analyzed using Enhanced BCA Protein Assay Kit (Beyotime). Subsequently, the proteins were separated using sodium dodecyl sulfate-polyacrylamide gel electrophoresis (SDS-PAGE) (Beyotime), followed by shifting on polyvinylidene fluoride (PVDF) membrane. After performing a blocking step using 5% non-fat milk, the membranes were incubated with the primary antibodies at 4℃ for an overnight period. Subsequently, they underwent a 2 h incubation at 37℃ with Goat Anti-Mouse IgG H&L (HRP) (ab205719, 1:10000, Abcam) or Goat Anti-Rabbit IgG H&L (HRP) (ab97051, 1:5000, Abcam). Ultimately, the results were determined using enhanced chemiluminescence detection. The information of the primary antibodies was as follows:β-actin (ab6276, 1:12000, Abcam), Msi1 (ab52865, 1:1000, Abcam), Sex Determining Region Y-Box 2 (SOX2) (ab97959, 1:1000, Abcam), octamer-binding transcription factor 4 (OCT4) (ab181557, 1:1000, Abcam), Hexokinase 2 (HK2) (ab209847, 1:1000, Abcam), Lactate dehydrogenase A (LDHA) (ab52488, 1:10000, Abcam), CD81 (ab79559, 1:1000, Abcam), CD9 (ab236630, 1:1000, Abcam), TSG101 (ab125011, 1:10000, Abcam), and Calnexin (ab22595, 1:1000, Abcam). Each experiment had three duplicates.

### Assessment of glucose uptake and lactate production

The assessment of cellular glucose uptake and lactate production was carried out. Glucose uptake in cells was measured using the Glucose Uptake Assay Kit from Beyotime, while lactate production was quantified with the Lactate Assay kit (BioVision, Inc., Milpitas, CA, USA). All experimental procedures strictly adhered to the guidelines provided by the manufacturers. Each experiment had three duplicates.

### Xenograft tumor models and immunohistochemistry (IHC)

BALB/C nude mice aged 6 weeks (Sibefu Biotechnology Co., Ltd., Beijing, China) were employed to establish xenograft tumor models. Shortly, the mice were injected subcutaneously with approximately 2 × 10^6^ SiHa cells, and then were placed in a normal environment to induce xenograft of tumor cells. Additionally, the exosomes were isolated from sh-NC or sh-Msi1-transfected M2 macrophages. In the group treated with exosomes, the mice were injected twice a week through the tail vein with 1 × 10^12^ exosome particles (suspended in 100 µL PBS). Control mice in this study were injected with an equal volume of PBS. Tumor volume was first measured on day 5 of exosome treatment and subsequently monitored at 5-day intervals until the endpoint on day 30, when all mice were euthanized. The tumor tissues were collected to analyze the Ki67, Msi1, SOX2, or HK2 expression through IHC. Animal experiments were allowed by Animal Care and Use Committee of the Affiliated Oncology Hospital, Xinjiang Medical University. Each group had six repetitions.

For IHC, 3 μm paraffin tissue sections were deparaffinized, rehydrated, and subjected to antigen retrieval by heating for 20 min at 97℃ and brief proteolytic digestion. Subsequently, endogenous peroxidases were blocked, and sections were incubated with anti-Ki67 (ab15580, 1:500, Abcam), anti-Msi1 (ab52865, 1:500, Abcam), anti-SOX2 (ab97959, 1:300, Abcam), or anti-HK2 (ab209847, 1:500, Abcam) overnight at 4℃. Next, slides were washed and combined with corresponding secondary antibody. Ultimately, the tissue sections underwent staining with diaminobenzidine (DAB) sourced from Huibaisi Biotechnology Co., Ltd. (Hunan, China). Subsequently, they were counterstained using hematoxylin and then observed. Each group had six repetitions.

### Statistical analysis

Statistical analyses were conducted utilizing GraphPad Prism software (GraphPad Software, Boston, MA, USA). The data were presented in the format of Mean ± standard deviation. For evaluating differences between two groups, Student’s t-test was employed. In contrast, when comparing three or more groups, analysis of variance (ANOVA) was used. A *P*-value below 0.05 was set as the criterion for statistical significance.

## Results

### Msi1 expression is associated with M2 macrophage polarization

**Fig. 1:**
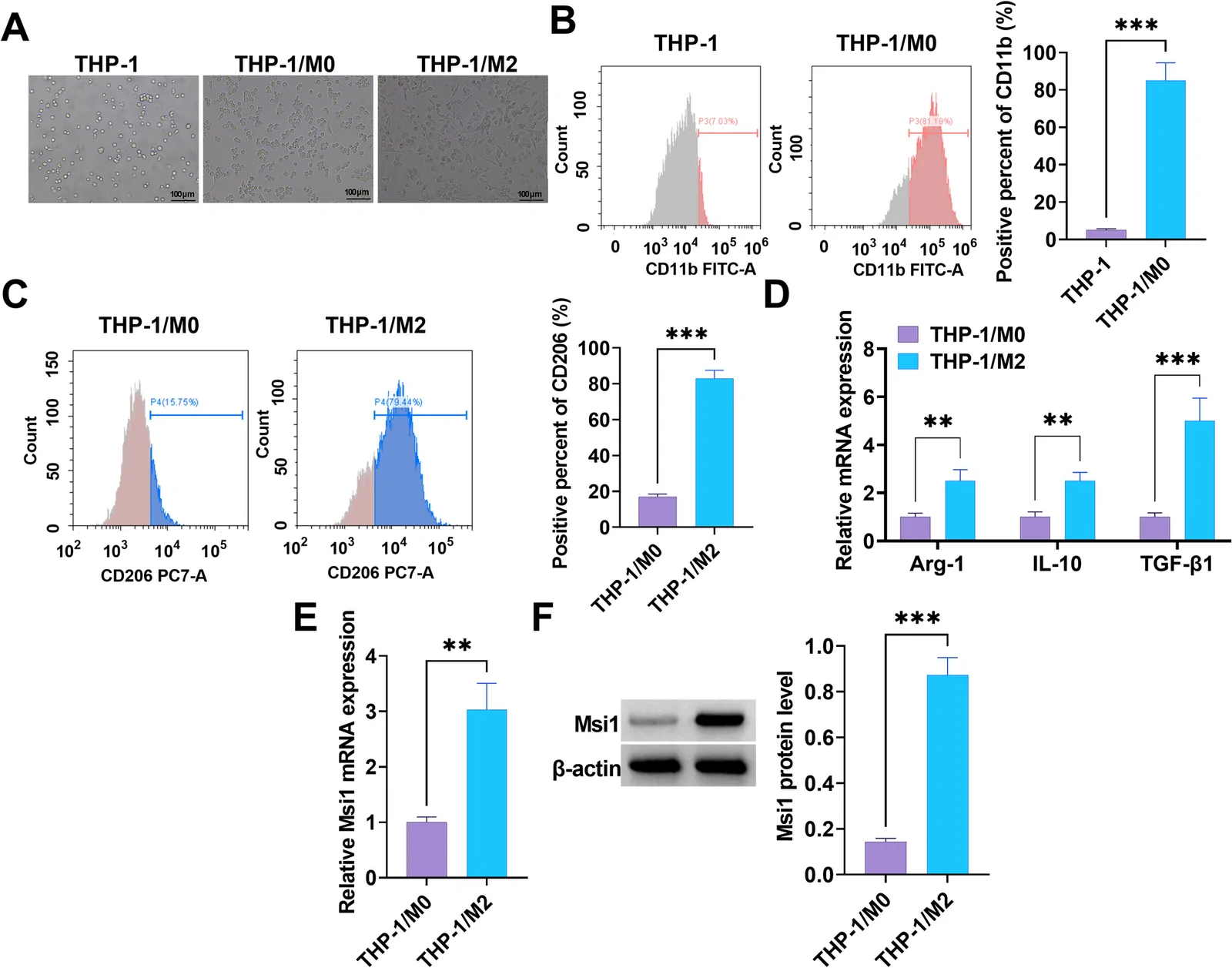
Msi1 expression is associated with M2 macrophage polarization. **A** Morphological changes in THP-1 cells at different stages during their induction into macrophages. **B** The positive percent of CD11b in THP-1 and THP-1/M0 groups was examined using flow cytometry. *** *P*<0.001 by Student’s *t*-test. **C** In THP-1/M0 and THP-1/M2 groups, the positive percent of CD206 was determined using flow cytometry. *** *P*<0.001 by Student’s *t*-test. **D** The Arg-1, IL-10, and TGF-β1 mRNA expression levels were analyzed using qRT-PCR. ** *P*<0.01 and *** *P*<0.001 by two-way ANOVA. **E**-**F** The Msi1 mRNA and protein levels were detected using qRT-PCR and western blot. ** *P*<0.01 and *** *P*<0.001 by Student’s *t*-test

The images of THP-1, THP-1/M0, and THP-1/M2 were shown in Fig. 1A. The positive percent of CD11b in THP-1/M0 group was increased when compared with THP-1 group (Fig. 1B). When compared with the THP-1/M0 group, the positive percent of CD206 was increased in THP-1/M2 group (Fig. 1C). As presented in Fig. 1D, M2 macrophage-associated factors Arg-1, IL-10, and TGF-β1 mRNA expression levels were up-regulated in THP-1/M2 group relative to THP-1/M0 group. Msi1 mRNA and protein expression levels were increased in THP-1/M2 compared to THP-1/M0 group (Fig. 1E F). Overall, Msi1 may modulate the polarization dynamics of M2 macrophages.

### M2 macrophages promote cervical cancer cell proliferation and invasion while inhibiting cell apoptosis partially through exosome secretion

**Fig. 2:**
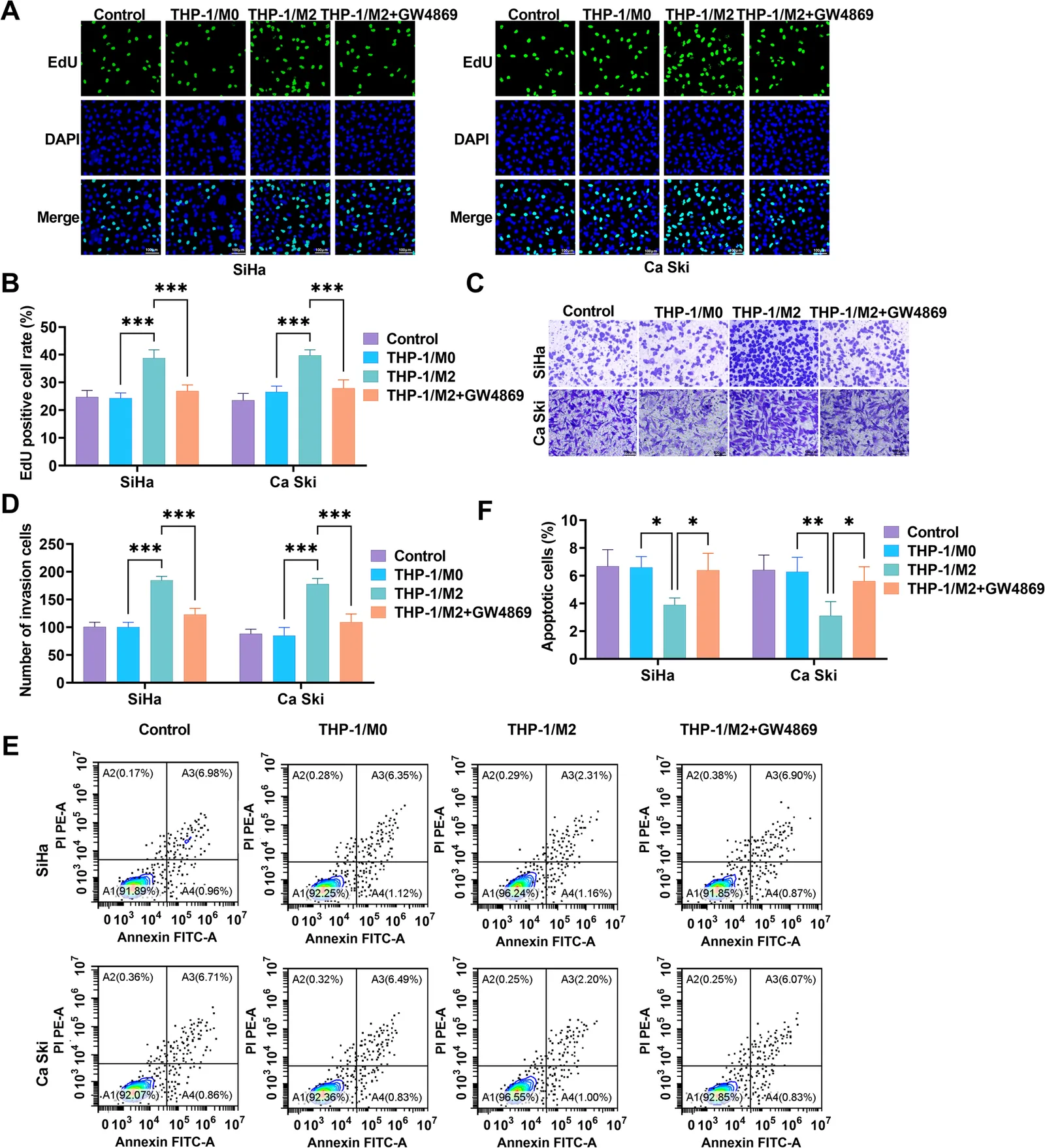
M2 macrophages promote cervical cancer cell proliferation and invasion as well as inhibit cell apoptosis. **A**-**F** SiHa and CaSki cells were co-cultured with THP-1/M0 or THP-1/M2. Additionally, GW4869 was used to block exosome generation. **A**-**B** The cell proliferation was analyzed using EdU staining. **C**-**D** Transwell assays were used to examine the cell invasion. **E**-**F** The cell apoptosis was determined using flow cytometry. * *P*<0.05, ** *P*<0.01, and *** *P*<0.001 by two-way ANOVA

To investigate whether M2 macrophages can regulate cervical cancer cell malignant behavior, THP-1/M0 cells were induced into M2 macrophages, and SiHa and Ca Ski cells were co-cultured with THP-1/M0 or THP-1/M2 macrophages. To block exosome production, cells were treated with GW4869. Co-culture with THP-1/M2 macrophages significantly promoted SiHa and Ca Ski cell proliferation and invasion, but this effect was attenuated by GW4869 (Fig. 2A-D). The inhibitory effect of THP-1/M2 macrophage co-culture on the apoptosis of SiHa and Ca Ski cells was also attenuated by GW4869 treatment (Fig. 2E-F). Taken together, M2 macrophages facilitate cervical cancer cell proliferation and invasion while inhibiting cell apoptosis partially via the secretion of exosomes.

### M2 macrophages promote glycolysis and stemness of SiHa and Ca Ski cells

**Fig. 3:**
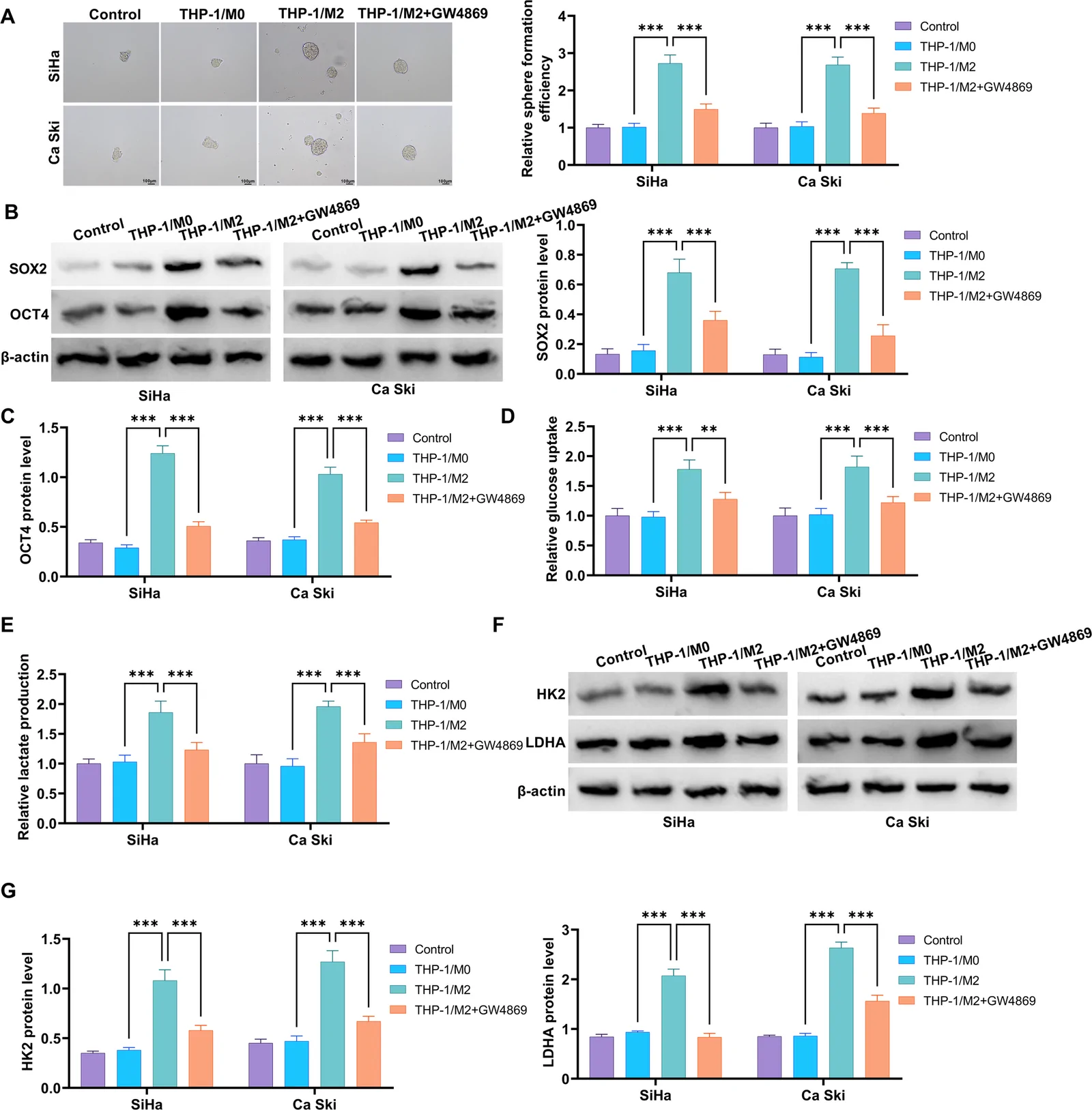
M2 macrophages enhance aerobic glycolysis and stemness of SiHa and Ca Ski cells via exosomes. **A**-**G** SiHa and Ca Ski cells were classified into four groups based on different treatments, named Control, THP-1/M0, THP-1/M2, and THP-1/M2 + GW4869 groups. **A** The sphere formation was analyzed using sphere formation assays. **B**-**C** Western blot was used to examine the SOX2 and OCT4 expression. **D**-**E** The glucose uptake and lactate production were determined using corresponding detection kits. **F**-**G** The HK2 and LDHA protein expression levels in each group were examined using western blot. ** *P*<0.01 and *** *P*<0.001 by two-way ANOVA

To investigate the effects of M2 macrophages on glucose metabolism and stemness of cervical cancer cells, the cervical cancer cells were divided into Control, THP-1/M0, THP-1/M2, and THP-1/M2 + GW4869 groups. As shown in Fig. 3A, tumor sphere formation was promoted in SiHa and Ca Ski cells co-cultured with THP-1/M2 macrophages, which was abrogated by GW4869. When compared with Control group, M0 macrophages did not affect the SOX2 and OCT4 protein expression in SiHa and Ca Ski cells, but SOX2 and OCT4 protein expression was up-regulated by M2 macrophages; when compared with the THP-1/M2 group, GW4869 inhibited SOX2 and OCT4 expression in SiHa and Ca Ski cells (Fig. 3B-C). M2 macrophages co-cultured enhanced glucose uptake and lactate production, and increased expression of HK2 and LDHA, while GW4869 weakened these effects (Fig. 3D-G). In summary, M2 macrophages induce aerobic glycolysis and stemness of SiHa and CaSki cells partially via exosomes.

### M2 macrophage-derived exosomes increase Msi1 expression

**Fig. 4:**
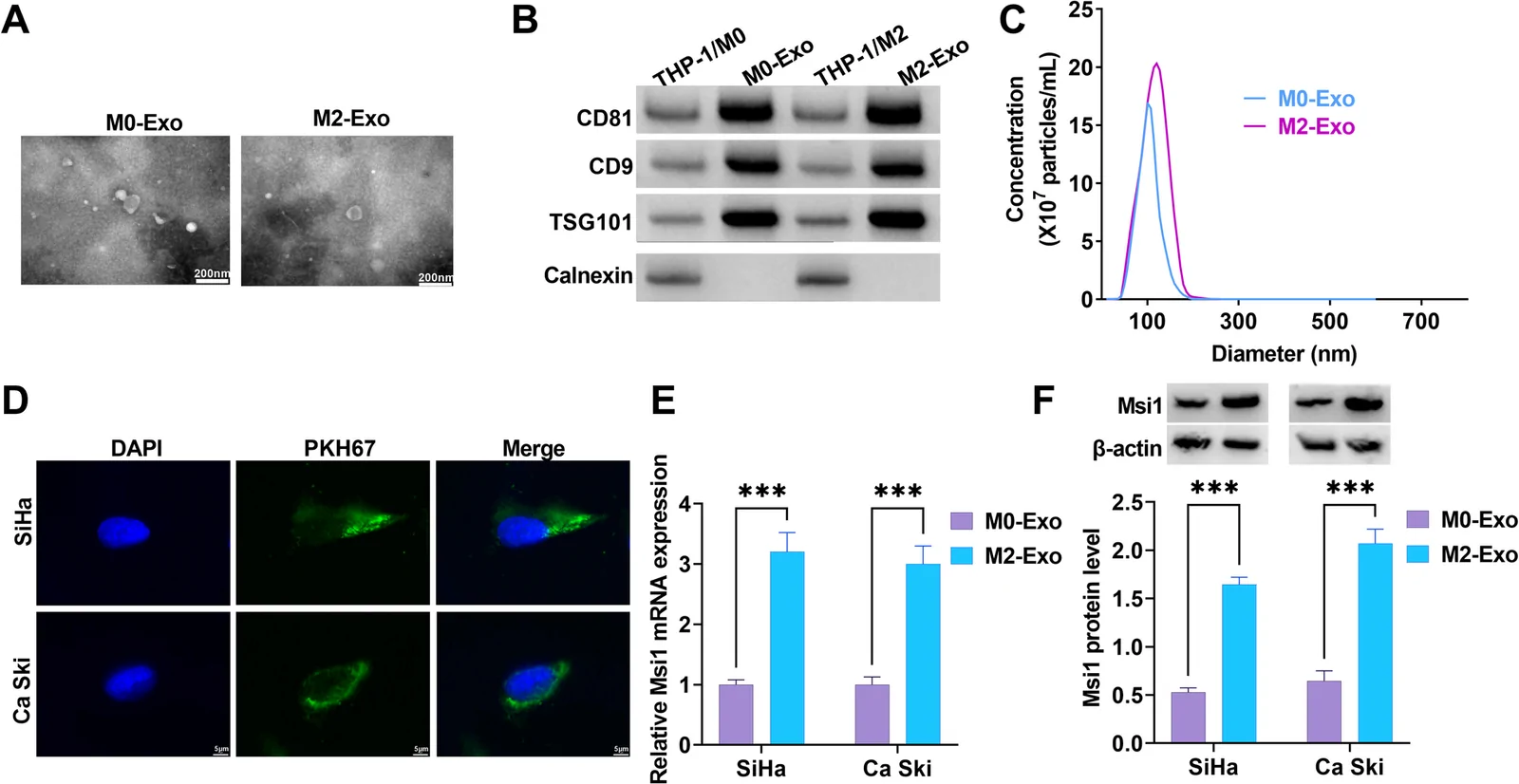
Identification of exosomes derived from M2 macrophages. **A** Transmission electron microscopy of exosomes in M2 macrophages. **B** Western blot was used to analyze the CD81, CD9, TSG101, and Calnexin expression. **C** Exosome size distribution as analyzed by NTA. **D** Exosome internalization by SiHa and Ca Ski cells was assessed using PKH67. **E**-**F** After SiHa and Ca Ski cells were treated with M2 macrophage-derived exosomes, the Msi1 mRNA and protein expression levels were examined using qRT-PCR and western blot. *** *P*<0.001 by two-way ANOVA

In order to further verify the effect of exosomes derived from M2 macrophages on the malignant progression of cervical cancer, the macrophage-derived exosomes were identified. The isolated exosomes exhibited typical cup-shaped morphology (Fig. 4A). Furthermore, CD81, CD9, and TSG101 protein expression levels were increased in comparison with THP-1/M0 or THP-1/M2 groups (Fig. 4B); additionally, in M0-Exo or M2-Exo group, the Calnexin was not expressed (Fig. 4B). The diameter of exosomes ranged from 50 to 200 nm (Fig. 4C). The results confirmed exosome enrichment. Cellular uptake assay showed that M2 macrophage-derived exosomes could be efficiently internalized by SiHa and Ca Ski cells (Fig. 4D). Notably, M2 macrophage-derived exosomes increased Msi1 mRNA and protein expression levels (Fig. 4E-F). In summary, exosomes are successfully isolated, and M2 macrophage-derived exosomes up-regulate Msi1 expression.

### M2 macrophage-derived exosomes promote malignancy through Msi1

**Fig. 5:**
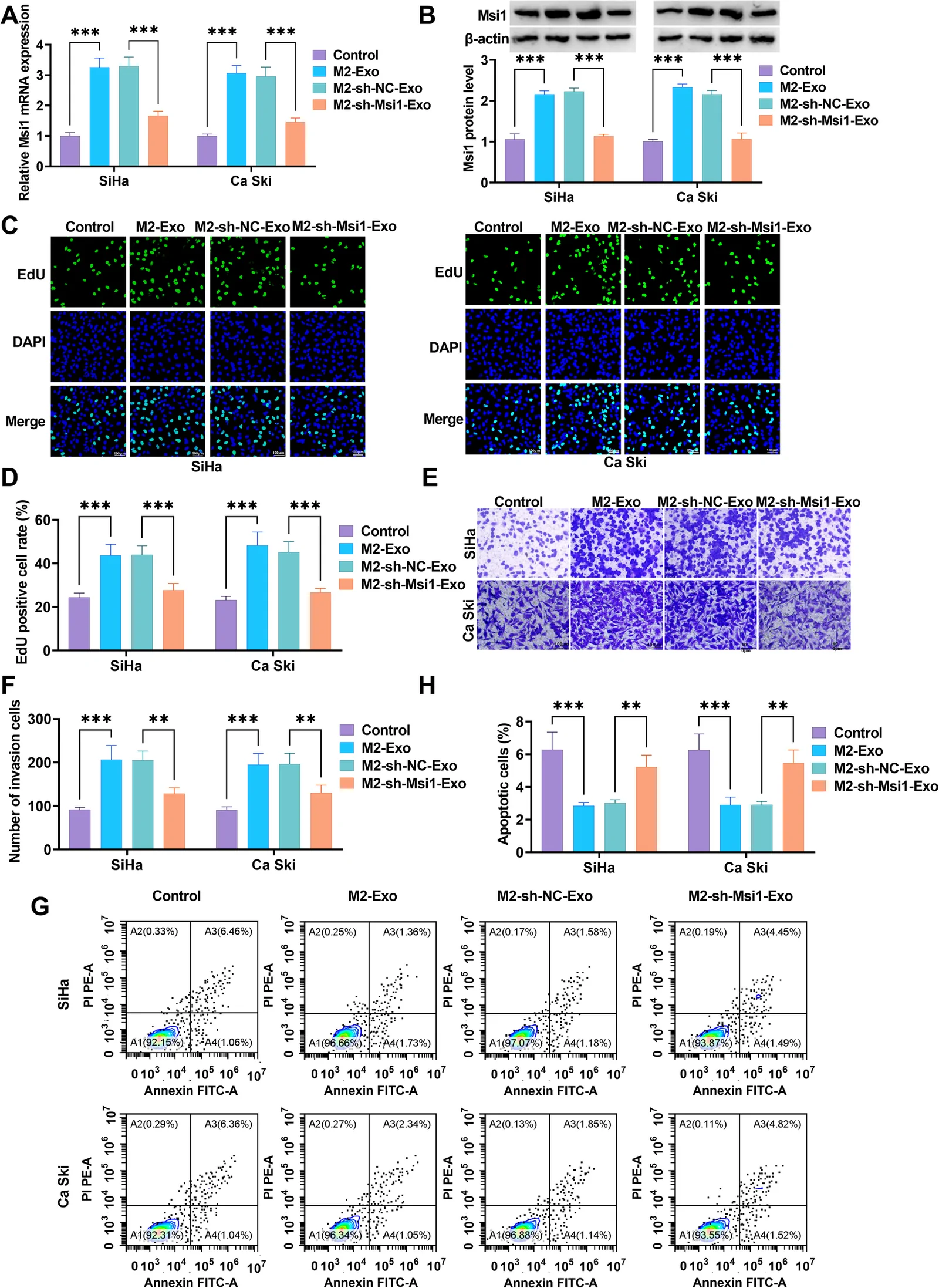
M2 macrophage-derived exosomes facilitate the malignancy by Msi1. The SiHa and Ca Ski cells were co-cultured with control, M2-Exo, M2-sh-NC-Exo, or M2-sh-Msi1-Exo, followed by the measurement of Msi1 mRNA and protein expression by qRT-PCR and western blot (**A**-**B**), SiHa and Ca Ski cell proliferation by EdU staining (**C**-**D**), cell invasion by Transwell (**E**-**F**), and cell apoptosis (**G**-**H**). ** *P*<0.01 and *** *P*<0.001 by two-way ANOVA

To determine whether Msi1 was associated with cervical cancer progression, this study first examined the expression of Msi1 in tissue samples. As shown in Fig. S1A-C, the Msi1 mRNA and protein expression levels in cervical squamous cell carcinoma (CESC) samples were elevated compared to Normal group. Correlation analysis between Msi1 and clinicopathological features is presented in Table 2. Additionally, the ROC curve suggested that Msi1 had potential diagnostic value (Fig. S1D). Therefore, the relationship between M2 macrophage-derived exosomes and Msi1 was analyzed. As shown in Fig. 5A and B, M2-Exo increased Msi1 mRNA and protein expression levels, which was abolished by M2-sh-Msi1-Exo. SiHa and Ca Ski cells co-cultured with M2-Exo or M2-shNC-Exo showed enhanced SiHa and Ca Ski cell proliferation and invasion with reduced apoptosis, whereas M2-sh-Msi1-Exo reversed these actions (Fig. 5C-H). Overall, Msi1 knockdown attenuated M2 macrophage-derived exosome-induced promotion of malignancy of SiHa and Ca Ski cells.

**Table 2:** Correlation analysis between Msi1 and clinicopathological characteristics (*N*=41)

Factor		Number of patient	High expression (*N* = 21)	Low expression (*N* = 20)	*P*
Age					0.155
	<50	19	12	7	
	≥ 50	22	9	13	
HPV infect					0.034
	Negative	16	12	4	
	Positive	25	9	16	
Tumor size (cm)					0.443
	>4	18	8	10	
	≤ 4	23	13	10	
lymph node metastasis					0.043
	Yes	21	14	7	
	No	20	7	13	
FIGO staging					0.013
	І/ІІ	26	9	17	
	ІІІ	15	12	3	

### Silencing Msi1 abolishes M2 macrophage-derived exosome-induced aerobic glycolysis and stemness of SiHa and CaSki cells

**Fig. 6:**
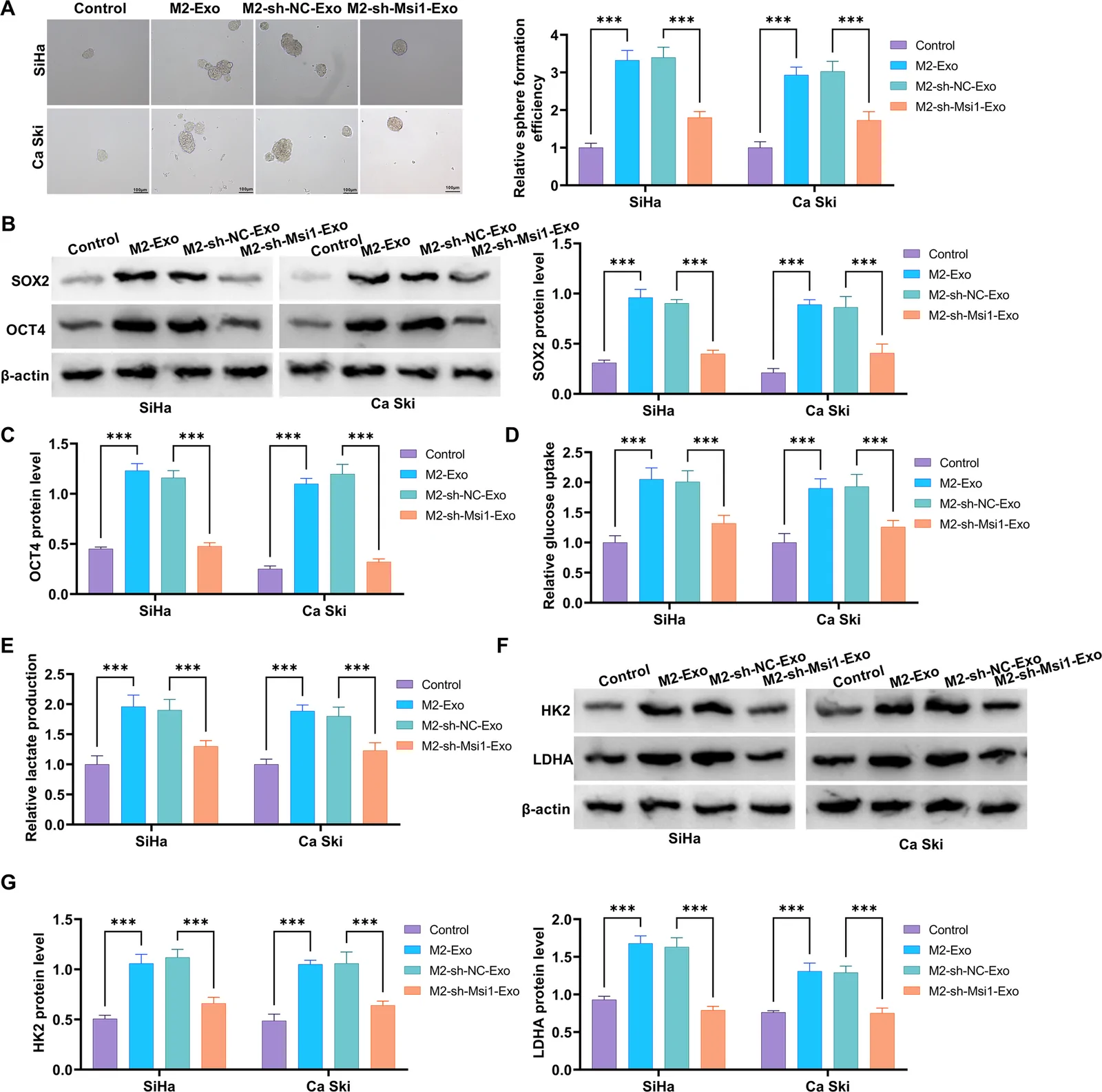
Msi1 knockdown abolishes M2 macrophage-derived exosome-induced aerobic glycolysis and stemness of SiHa and CaSki cells. **A**-**G** SiHa and Ca Ski cells were co-cultured with control, M2-Exo, M2-sh-NC-Exo, or M2-sh-Msi1-Exo. **A** The sphere formation was analyzed using sphere formation assays. **B**-**C** SOX2 and OCT4 expression levels were determined using western blot. **D**-**E** The glucose uptake and lactate production were examined by corresponding detection kits. **F**-**G** The HK2 and LDHA protein expression levels were detected using western blot. *** *P*<0.001 by two-way ANOVA

Researchers then examined the role of exosomal Msi1-mediated glucose metabolism and stemness of cervical cancer cells. SiHa and Ca Ski cells co-cultured with M2-Exo or M2-sh-NC-Exo demonstrated enhanced sphere formation and up-regulated SOX2 and OCT4 expression, while M2-sh-Msi1-Exo abrogated these actions (Fig. 6A-C). M2 macrophage-derived exosomes facilitated glucose uptake and lactate production in SiHa and Ca Ski cells, but these effects were weakened by M2-sh-Msi1-Exo (Fig. 6D-E). Furthermore, the expression of HK2 and LDHA was increased in SiHa and Ca Ski cells co-cultured with M2-Exo or M2-sh-NC-Exo, which was abrogated by M2-sh-Msi1-Exo (Fig. 6F-G). To sum up, exosomes derived from M2 macrophages carrying the Msi1 gene induce aerobic glycolysis and stemness of SiHa and CaSki cells.

### M2 macrophage-derived exosomal Msi1 accelerates cervical cancer progression in vivo

**Fig. 7:**
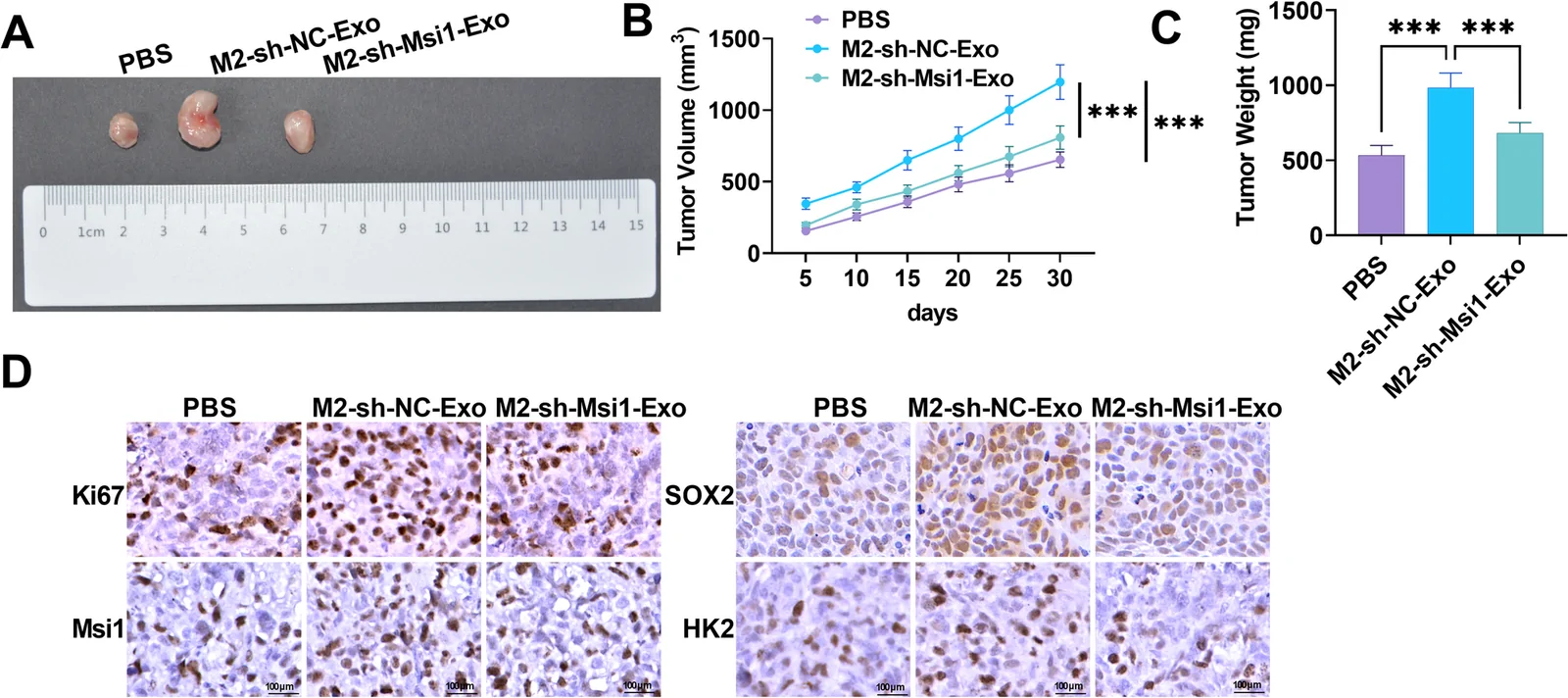
In vivo, Msi1 knockdown abolishes M2 macrophage-derived exosome-induced cervical cancer progression. Mice were injected subcutaneously with approximately 2 × 10^6^ SiHa cells and administered with control (PBS), M2-sh-NC-Exo, or M2-sh-Msi1-Exo. **A** The cervical cancer images were observed. **B** Tumor volume was shown. *** *P*<0.001 by two-way ANOVA. **C** Analysis of tumor weight. *** *P*<0.001 by one-way ANOVA. **D** The Ki67, Msi1, SOX2, and HK2 expression levels were examined using IHC assay. *** *P*<0.001

To further validate our in vitro findings, cervical cancer xenograft mouse models were established to analyze the role of M2 macrophage-derived exosomal Msi1. M2-sh-NC-Exo promoted tumor growth, an effect attenuated by M2-sh-Msi1-Exo (Fig. 7A-C). Additionally, the IHC results showed that M2-sh-NC-Exo increased Ki67, Msi1, SOX2, and HK2 expression, but these actions were reversed by M2-sh-Msi1-Exo (Fig. 7D). In summary, Msi1 knockdown abrogates M2 macrophage-derived exosome-induced cervical cancer progression in vivo.

## Discussion

Cervical cancer is a prevalent malignancy threatening women’s health, arising primarily from persistent high-risk HPV infection [26]. Although significant progress has been made in current therapeutic strategies for cervical cancer, the long-term prognosis for patients with recurrent or metastatic disease remains poor [27]. Consequently, elucidating the molecular mechanisms underlying cervical carcinogenesis is of critical clinical importance.

Macrophages are innate immune cells that play a significant role in both inflammatory responses and tumor immunity [28]. Among these, the M2 subtype has relatively weak antigen-presenting capacity and low anti-tumor activity, thereby promoting tumor growth and invasion [29]. The oncogenic protein CKLF-like MARVEL transmembrane domain-containing 6 drives M2a macrophage formation and promotes the progression of cervical cancer [30]. Notably, some studies have linked Msi1 to macrophage activation and polarization, as it regulates cytokine secretion and gene expression by regulating them, thereby creating a tumor-friendly microenvironment [31, 32]. Consistent with these findings, our data demonstrated that Msi1 expression was significantly up-regulated in M2 macrophages, suggesting a potential association between Msi1 and M2 macrophage polarization that warrants further investigation.

Exosomes play a crucial role not only in facilitating intercellular communication among cancer cells but also in mediating interactions between cancer cells and immune cells [33]. Importantly, exosomes show strong potential as biomarkers for the diagnosis of cervical cancer [34]. In our study, M2 macrophages promoted cervical cancer cell proliferation and invasion while reducing apoptosis, effects that were reversed by GW4869, indicating that exosome secretion is essential for these effects. It is well known that aerobic glycolysis, also known as the Warburg effect, is a hallmark of malignant tumors [35, 36]. Furthermore, emerging research has indicated that metabolic alterations occurring in cancer cells play a role in driving the initiation and advancement of cancer [37]. Our data showed that M2 macrophages promote aerobic glycolysis and stemness in cervical cancer cells, effects attenuated by GW4869. These findings aligning with previous reports in other tumors and provide new evidence in the context of cervical cancer, demonstrating that exosome release is a key mechanism by which M2 macrophages influence cervical cancer cell metabolism and stemness. To further confirm the involvement of exosomes, this study used GW4869 to block exosome generation, which abolished M2 macrophage-induced cervical cancer progression. This observation strongly suggests that M2 macrophages promote cervical cancer progression primarily through exosome secretion. Additionally, our data showed that Msi1 expression was increased in M2 macrophage-derived exosomes.

Msi1 is a key regulator of stem cell activity [38], and an established oncogenic driver across malignant malignancies [39]. For instance, Msi1 enhanced prostate cancer cell proliferation, migration and glycolysis via facilitating ABHD2 Transcription [21]. In osteogenic contexts, it stabilizes MACF1 to suppress high glucose-induced apoptosis in MC3T3-E1 cells and facilitates osteogenic differentiation via the Wnt/β-catenin pathway [40]. Furthermore, Msi1 enhances tumor invasion through Vascular Cell Adhesion Molecule 1, and regulates the excessive activation of the DDR by increasing homologous recombination repair while avoiding apoptosis, thereby modulating the radioresistance of glioblastoma [41]. Specifically in cervical cancer, Msi1 has been reported to promote tumor progression and cellular proliferation by regulating the cell cycle checkpoint proteins p21, p27, and p53 [24], and to enhance tumor progression through epithelial-mesenchymal transition [25]. Consistent with these previous reports, our data showed that Msi1 expression was elevated in CESC samples, with ROC curve analysis indicating a potential diagnostic value that should be interpreted cautiously given the limited sample size. Based on these observations, our study hypothesized that M2 macrophage-derived exosomes may influence cervical cancer progression by transferring Msi1. To test this hypothesis, our study silenced Msi1 in M2 macrophages and observed that the pro-malignant effects of M2 macrophage-derived exosomes on SiHa and CaSki cells were substantially abolished. Moreover, exosomes derived from M2 macrophages carrying Msi1 promoted aerobic glycolysis and stemness in cervical cancer cells. These loss-of-function experiments provide evidence supporting a causal role for exosomal Msi1 in mediating these effects. The consistency between our in vivo and in vitro findings strengthens the validity of these conclusions.

The present study demonstrated that M2 macrophage-derived exosomes deliver Msi1 to cervical cancer cells, leading to enhanced glycolysis (increased HK2 and LDHA expression, glucose uptake, and lactate production) and stemness (upregulated SOX2 and OCT4). However, the precise molecular mechanisms by which Msi1 drives these metabolic and stemness changes remain to be fully elucidated. Msi1 is an RNA-binding protein that primarily functions by stabilizing target mRNAs or regulating their translation [21, 42]. In the context of metabolic regulation, Msi1 has been reported to promote glycolysis in prostate cancer by facilitating ABHD2 transcription [21]. Furthermore, the existing literature suggests potential connections between Msi1 and several key oncogenic signaling pathways, including c-Myc, HIF-1α, and PI3K/AKT, all of which are well-established drivers of aerobic glycolysis and cancer stemness [43–45]. For instance, HK2 and LDHA, two key glycolytic enzymes, are known transcriptional targets of c-Myc and HIF-1α [46, 47], while SOX2 and OCT4 are core pluripotency factors whose expression can be modulated by PI3K/AKT signaling [48]. It is therefore plausible, as a hypothesis for future investigation, that exosomal Msi1 may promote glycolysis and stemness in cervical cancer cells through post-transcriptional stabilization or enhanced translation of mRNAs encoding components of these pathways. Nevertheless, the current study did not experimentally validate these downstream mechanisms.

However, this study has several limitations. First, although exosome markers were validated and particle number was normalized, the use of density gradient ultracentrifugation would provide higher purity in future studies. Second, functional experiments were limited to two cervical cancer cell lines (SiHa and CaSki); validation in additional lines and primary patient-derived cells is needed to confirm the generalizability of the findings. Third, while this study demonstrated that Msi1 is essential for M2 macrophage-derived exosome-mediated pro-tumorigenic effects, it is acknowledged that the precipitation-based exosome isolation method raises concerns regarding purity, and additional validation (e.g. via ultracentrifugation or density gradient) would improve experimental rigor. Furthermore, the downstream molecular targets of Msi1 (particularly the direct mRNA targets responsible for upregulating HK2, LDHA, SOX2, and OCT4) remain unidentified. The potential involvement of signaling pathways such as c-Myc, HIF-1α, or PI3K/AKT also lacks experimental validation. The discussion of these pathways in the preceding section is speculative and intended to generate hypotheses rather than draw definitive conclusions. Rescue experiments (e.g. overexpression of putative downstream effectors or inhibition of candidate pathways) are necessary to establish causal relationships and represent an important direction for future investigations. Fourth, the xenograft mouse model does not fully capture the complexity of the human tumor microenvironment, including interactions with endogenous immune cells. Fifth, regarding clinical relevance, the primary objective of the clinical sample analysis was to validate Msi1 expression differences between cervical cancer and normal tissues, not to establish a prognostic model. The patient cohort was relatively small (*N* = 41) and lacked sufficient follow-up data for survival or multivariate analysis; therefore, no prognostic conclusions can be drawn from this dataset. The ROC analysis is presented as a descriptive finding of diagnostic potential that requires validation in larger, independent cohorts. Any language suggesting prognostic value has been intentionally avoided, and clinical conclusions remain limited to the observed expression differences. Finally, larger clinical cohorts with complete follow-up data are needed to assess the correlation between M2 macrophage-derived exosomal Msi1 levels and patient prognosis or therapeutic response.

In summary, this study demonstrates that M2 macrophage-derived exosomes deliver Msi1 to promote cervical cancer progression and aerobic glycolysis. While exosomal Msi1 emerges as a key mediator of M2-induced tumor promotion, these conclusions are preliminary given the unresolved downstream mechanisms and small clinical cohort, necessitating further mechanistic and clinical validation.

## Supplementary Information

Supplementary Material 1. Figure S1 Expression of Msi1 in tissue samples. (A) The expression of Msi1 mRNA in tissue samples using qRT-PCR (N (normal)=41 and N (CESC)=41). *** *P*＜0.001 by Student’s t-test. (B-C) The protein expression of Msi1 was analyzed using western blot (N=5). *** *P*＜0.001 by Student’s t-test. (D) A ROC curve was used to discriminate two distinct groups of Msi1 expression. *** *P*＜0.001.

Supplementary Material 2.

## Data Availability

The analyzed data sets generated during the present study are available from the corresponding author on reasonable request.
