# Supplementary material for: M2 macrophage-derived exosomes promote the malignant progression and glycolysis of cervical cancer via Msi1
Source: Hereditas. 2026 Jun 25;163:116. doi: 10.1186/s41065-026-00703-9 (PMC13678903; doi:10.1186/s41065-026-00703-9)

fig 1F
1:THP-1/M0
2:THP-1/M2

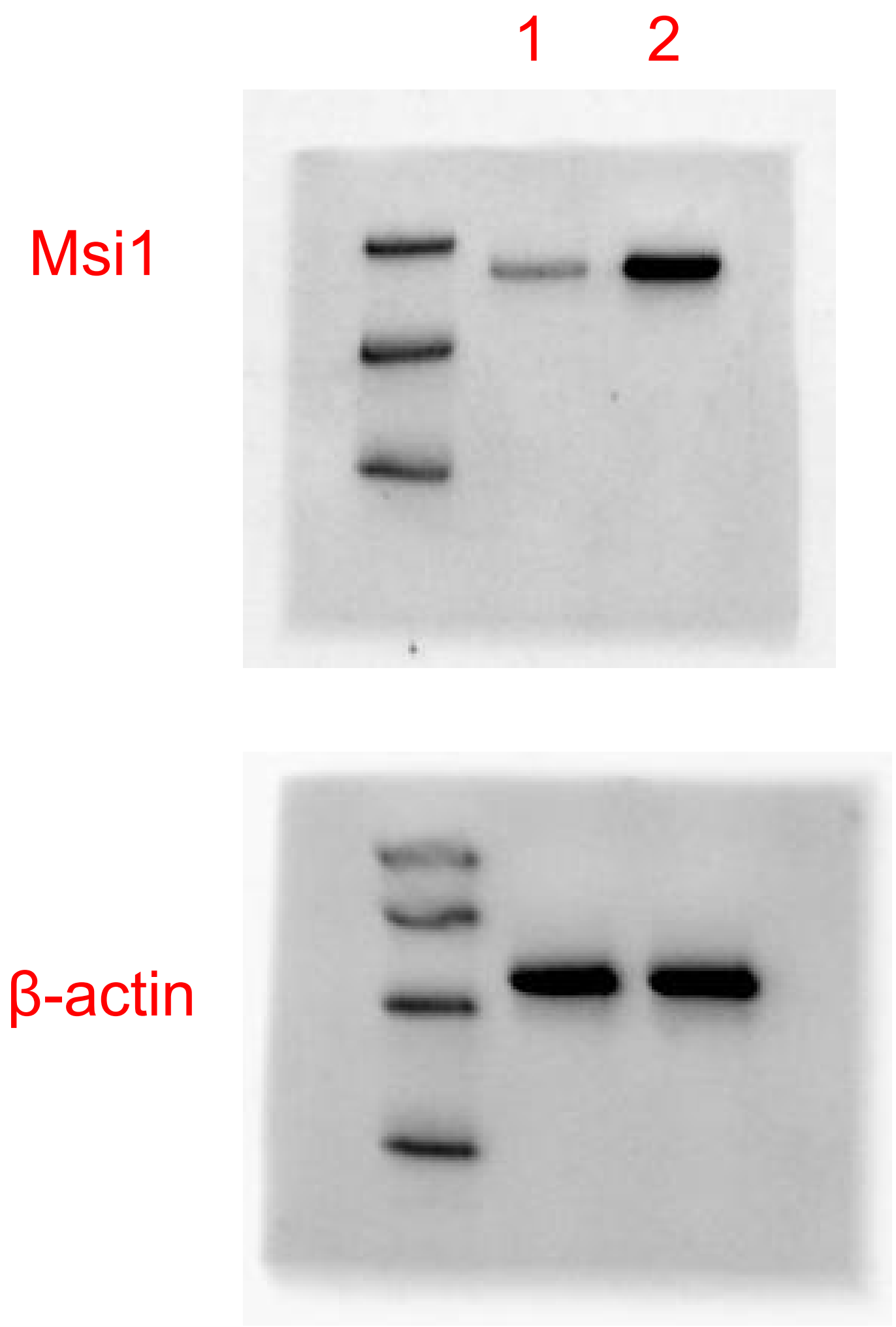

fig 3B
1:Control
2:THP-1/M0
3:THP-1/M2
4:THP-1/M2+GW4869

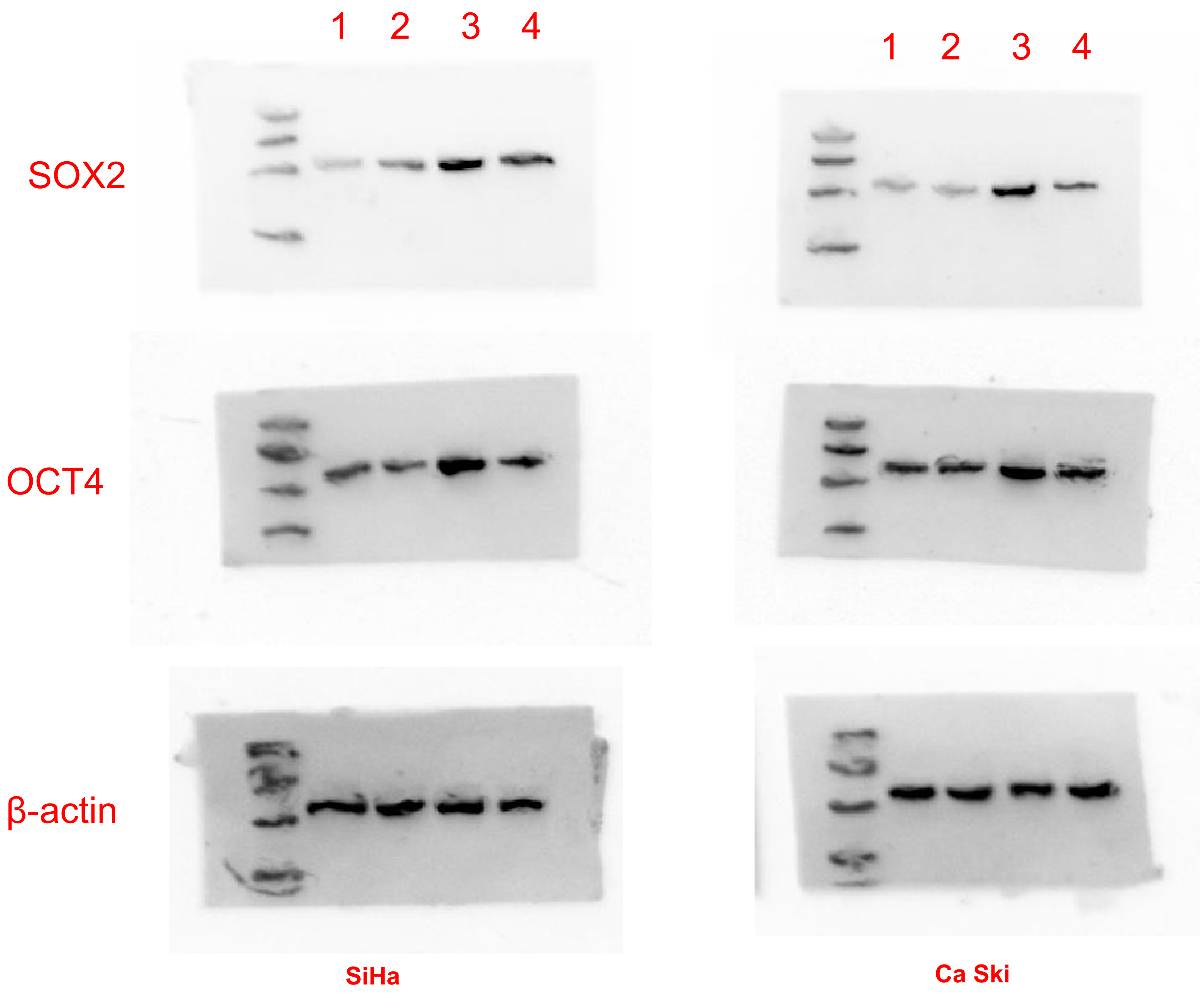

fig 3F
1:Control
2:THP-1/M0
3:THP-1/M2
4:THP-1/M2+GW4869

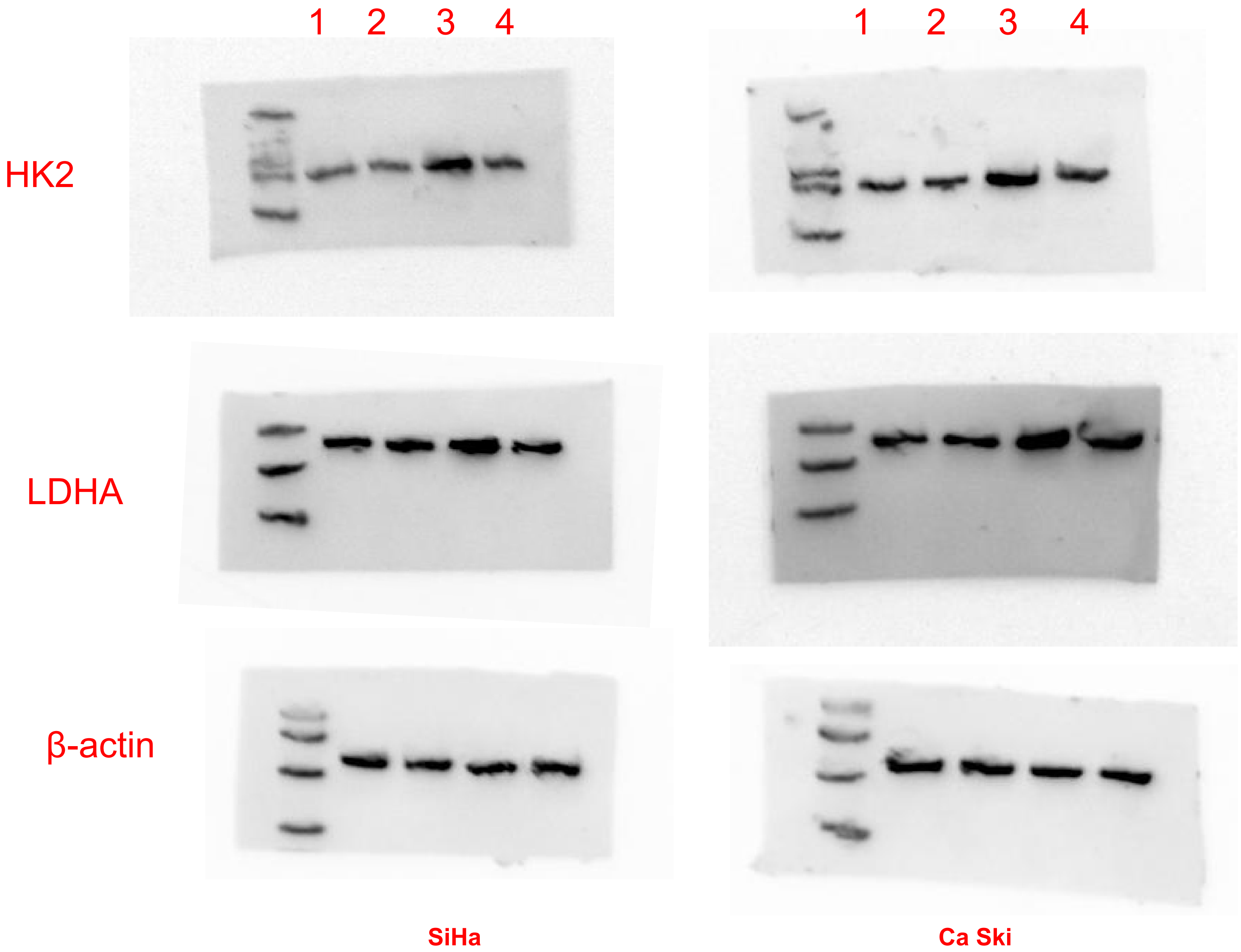

fig 4B
1:THP-1/M0
2:M0-Exo
3:THP-1/M2
4:M2-Exo

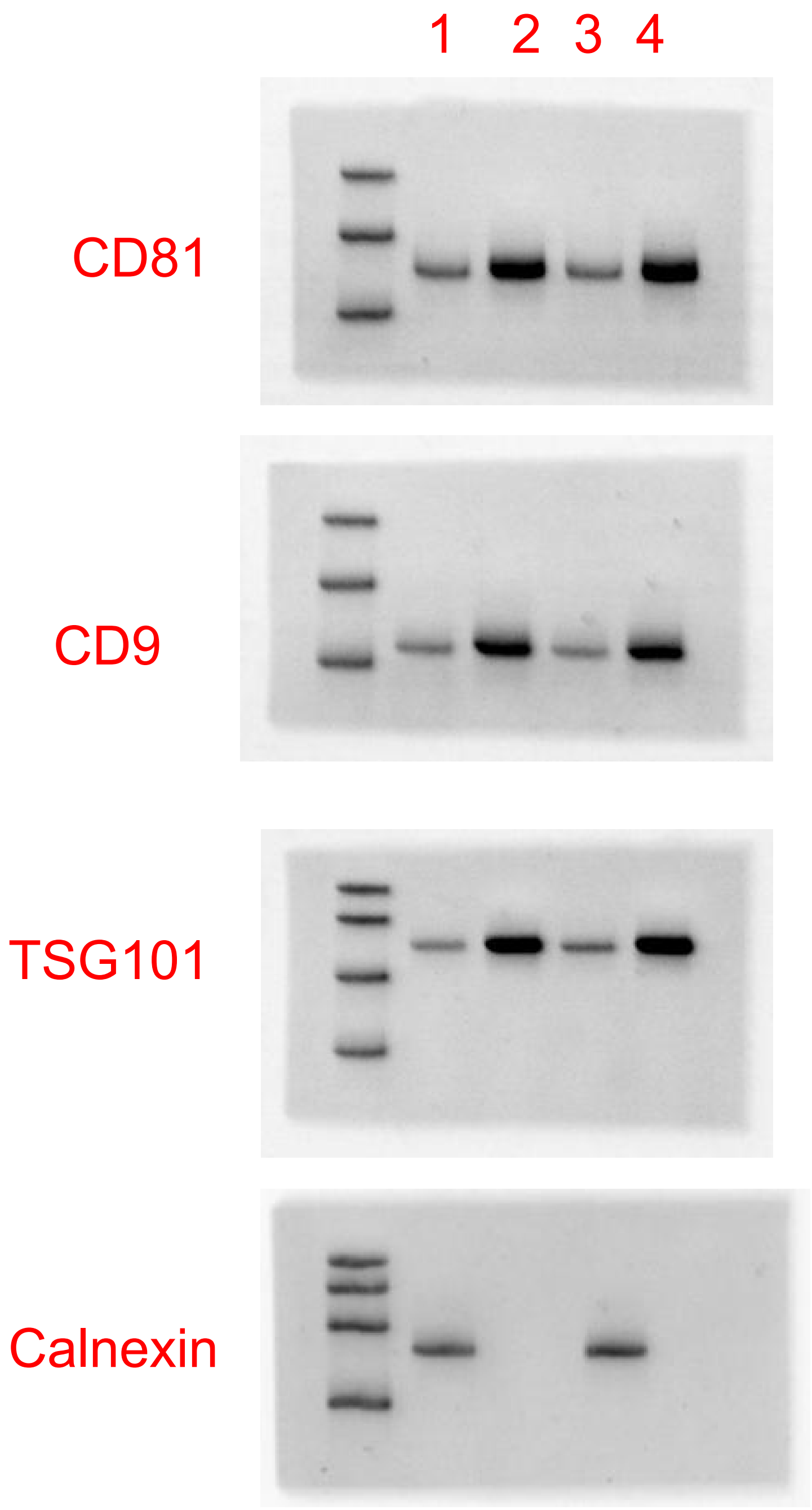

fig 4F
1:M0-Exo
2:M2-Exo

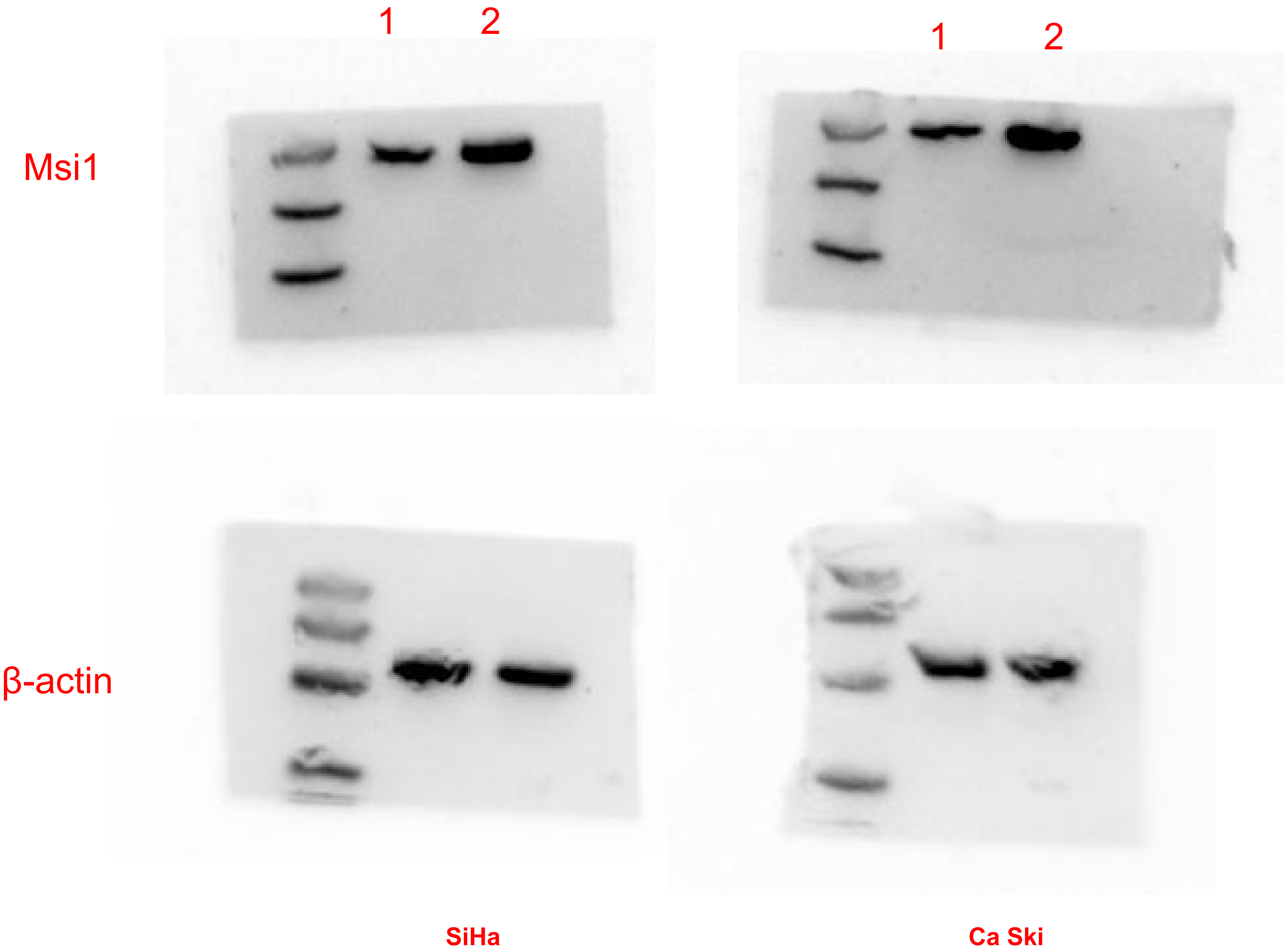

fig 5B
1:Control
2:M0-Exo
3:M2-sh-NC-Exo
4:M2-sh-Msi1-Exo

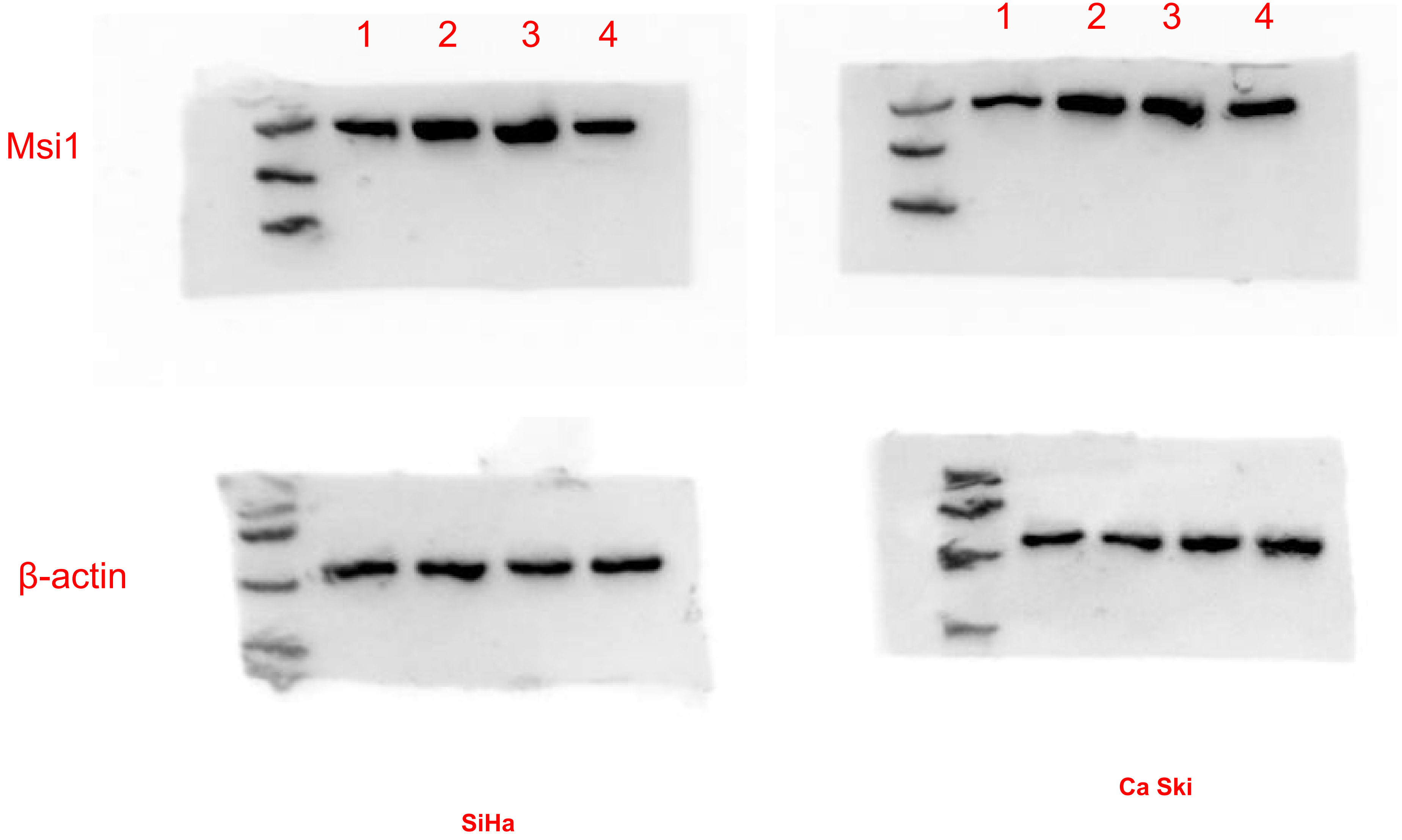

fig 6B
1:Control
2:M0-Exo
3:M2-sh-NC-Exo
4:M2-sh-Msi1-Exo

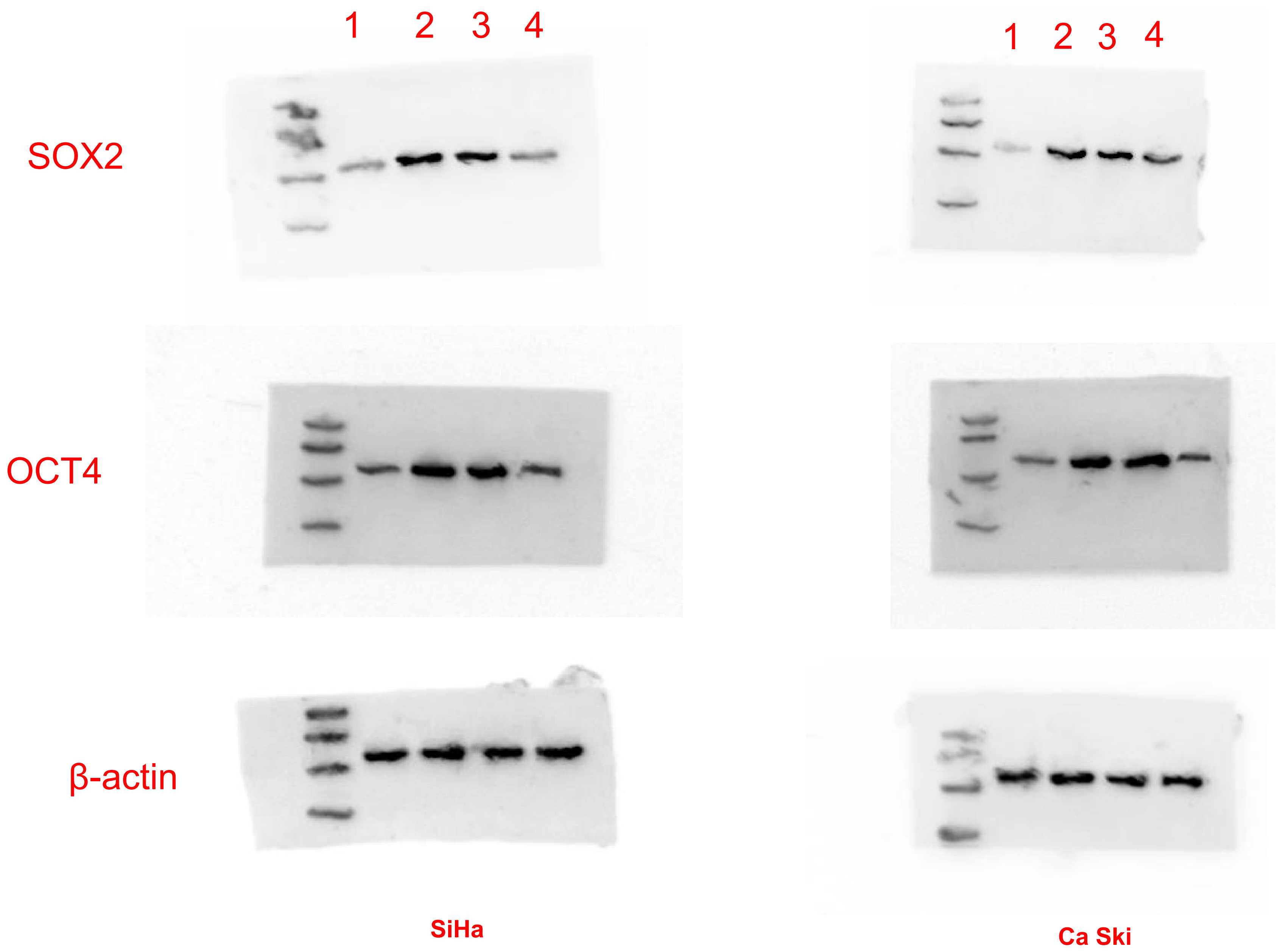

fig 6F

1:Control
2:M0-Exo
3:M2-sh-NC-Exo
4:M2-sh-Msi1-Exo

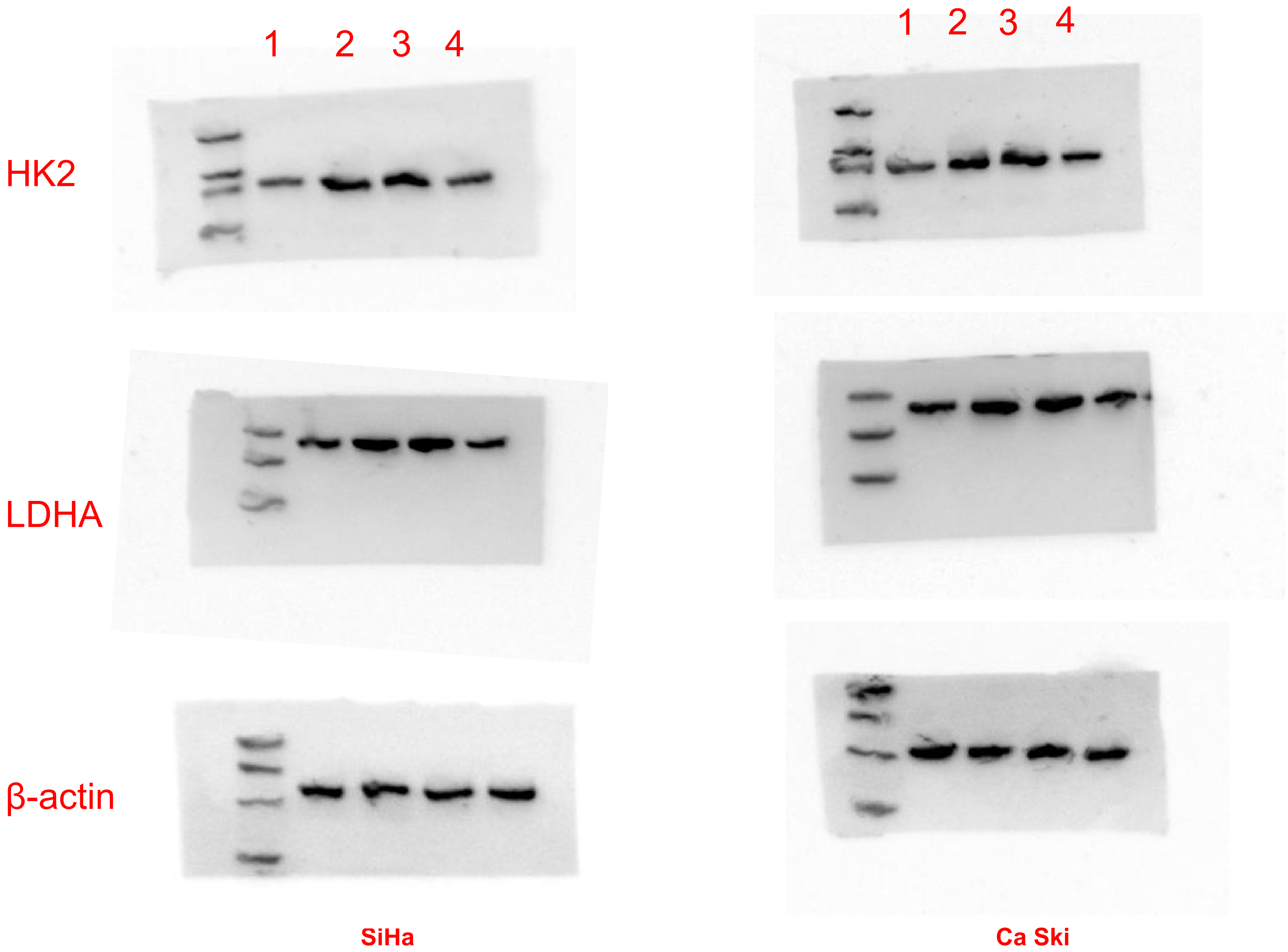

fig S1B
1:Normal
2:CESC

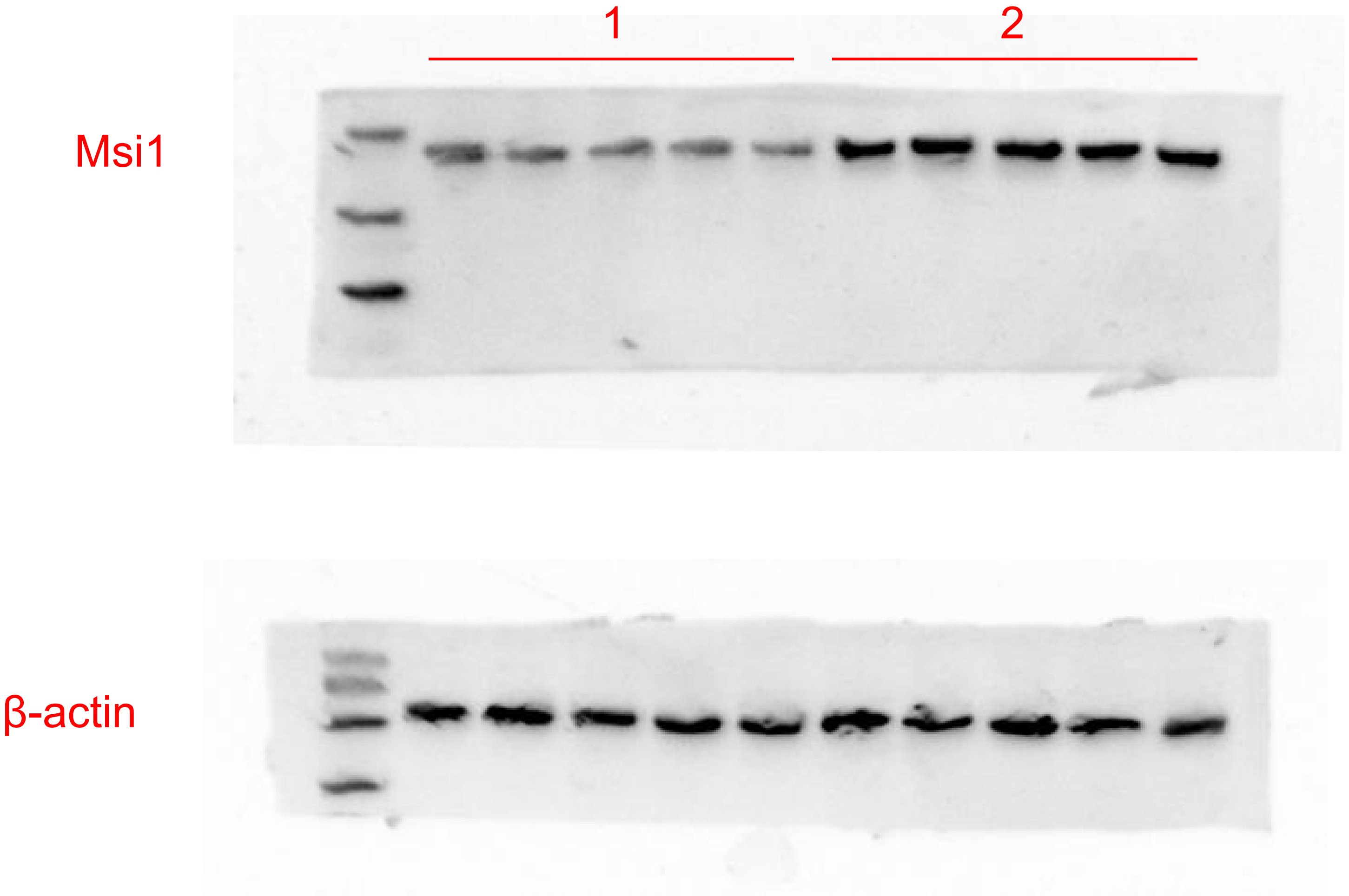

Supplement: Supplementary file 1 [file 41065_2026_703_MOESM2_ESM.pdf]
